# Targeted prostate biopsy using a cognitive fusion of multiparametric magnetic resonance imaging and transrectal ultrasound in patients with previously negative systematic biopsies and non-suspicious digital rectal exam

**DOI:** 10.3325/cmj.2020.61.49

**Published:** 2020-02

**Authors:** Tomislav Kuliš, Toni Zekulić, Ana Marija Alduk, Mario Lušić, Stela Bulimbašić, Vladimir Ferenčak, Ivica Mokos, Tvrtko Hudolin, Željko Kaštelan

**Affiliations:** 1 Department of Urology, University Hospital Center Zagreb, Zagreb, Croatia; 2 University of Zagreb School of Medicine, Zagreb, Croatia; 3 Department of Radiology, University Hospital Center Zagreb, Zagreb, Croatia; 4 Department of Pathology, University Hospital Center Zagreb, Zagreb, Croatia

## Abstract

**Aim:**

To compare cognitive fusion targeted and systematic prostate biopsy in patients with repeated negative systematic biopsy but persistent clinical suspicion for prostate cancer.

**Methods:**

The study enrolled 63 patients with at least one previously negative systematic biopsy who underwent targeted prostate biopsy using multiparametric magnetic resonance imaging (mpMRI) and transrectal ultrasound (TRUS) in addition to standardized systematic biopsy from July 2016 to May 2018. Multiparametric MRI was performed with 3 Tesla device by uro-radiologists experienced in prostate cancer. Lesions with Prostate Imaging Reporting and Data System 3, 4, and 5 were considered suspicious. Targeted biopsies were performed with cognitive fusion of TRUS and mpMRI.

**Results:**

Prostate cancer detection, using either targeted or systematic biopsy, was 60.32%. Targeted biopsies were positive in 52.38% and systematic biopsies in 47.62% of patients. The median highest percentage of cancer involvement per biopsy core was significantly higher in targeted cylinders. The biopsies obtained by using the two techniques did not significantly differ in Gleason score.

**Conclusion:**

Cognitive targeted prostate biopsy based on mpMRI presents a valuable addition to systematic biopsy in patients with repeated negative systematic biopsies but persistent clinical suspicion of prostate cancer.

Prostate cancer is the second most common malignant neoplasm and the fifth leading cause of death in the male population, with the expected increase in incidence and mortality due to global population aging (1). In Croatia, it is the second most common cancer in terms of incidence and mortality (2). Patients with clinical suspicion of prostate cancer (prostate specific antigen [PSA]>4.0 ng/mL, abnormal digital rectal exam) undergo systematic transrectal ultrasonography (TRUS)-guided biopsy, during which 10 to 12 cores of prostate tissue are obtained for pathohistological evaluation (3). Such non-targeted, systematic biopsy may overdiagnose insignificant cancer and underdiagnose clinically significant cancer, particularly cancers located in the apical and anterior prostate zones (4-6). The overdiagnosis of clinically insignificant prostate cancer leads to overtreatment, higher psychological burden, and treatment complications, which all results in increased health care system expenses.

In recent years, multiparametric magnetic resonance imaging (mpMRI) has shown promise as a diagnostic tool for prostate cancer. The combination of anatomic, T1, T2, functional, diffusion weighted, and dynamic contrast enhance imaging provides good sensitivity for the detection and localization of tumors with the Gleason score ≥7 (7,8). Targeted biopsies based on mpMRI are more likely to help diagnose clinically significant prostate cancer and reduce the number of diagnoses of clinically insignificant cancers compared with systematic biopsies, thus reducing the number of overtreated patients and treatment costs (9-11). In PROMIS study, when compared with TRUS biopsy, mpMRI-based targeted biopsy had a higher sensitivity and negative predictive value for diagnosing clinically significant prostate cancer (9).

Patients with a negative systematic biopsy but with present clinical suspicion for prostate cancer due to elevated PSA levels present an unresolved diagnostic challenge. The repeat biopsy after a negative first systematic biopsy using TRUS was positive for prostate cancer in about 30% of patients, a substantially lower rate compared with that of saturation biopsy (12,13). In another study, the first systematic biopsy controlled by TRUS was positive in 20% of patients, while the fourth biopsy was positive in only 4% of patients (14). Due to the poor results of repeated systematic biopsies, these patients are recommended to undergo a targeted biopsy with mpMRI (15).

The aim of this study is to compare cognitive fusion targeted and systematic prostate biopsy in patients with repeated negative systematic biopsy but persistent clinical suspicion for prostate cancer.

## Patients and methods

### Patients

We prospectively enrolled 63 male patients treated at the Department of Urology, University Hospital Center Zagreb from July 2016 to May 2018. The patients underwent cognitive targeted prostate biopsy by using mpMRI and TRUS after at least one previously negative systematic biopsy. Power analyses based on the data from previous studies yielded 80% power and alpha value of 0.05, indicating that 39 patients were needed to show the difference between targeted and systematic biopsy. Inclusion criteria were still present clinical suspicion of prostate cancer due to constantly elevated or rising PSA (>4 ng/mL) and PI-RADS 3-5 lesion identified using mpMRI. The study was approved by the Ethics Committee of Zagreb University Hospital Center (02/21 AG).

### MRI specification and technique

All MRI examinations were performed with a 3T Siemens Prisma scanner (Siemens Medical Solutions, Erlangen, Germany) by using a phased array coil. T2-weighted images were taken in the sagittal, coronal, and transversal planes with the 200 mm ×200 mm field-of-view, 3-mm slice thickness, repetition time of 6000-6620 ms, and echo time of 91-101 ms. Diffusion-weighted sequences were performed with the b values of 0 s/mm^2^, 500 s/mm^2^, 1500 s/mm^2^, and 2000 s/mm^2^. Dynamic sequences consisted of T1-weighted fat saturated images with 320 mm ×206 mm FOV, 3-mm slice thickness, TR 4 ms, TE 1 ms, before and 50 times after the administration of 0.2 mL/kg gadopentetate dimeglumine (Dotarem, Guerbet S.A., France) at a concentration of 0.5 mmol/mL. All mpMRI findings were interpreted by uro-radiologists experienced in prostate cancer. According to the version 2 of Prostate Imaging Reporting and Data system (PI-RADS), lesions with PI-RADS 3, 4, and 5 on mpMRI were considered suspicious (16).

### Biopsy technique and pathohistological evaluation

All targeted and systematic biopsies were performed by the same urologist with an ultrasound station (Flex Focus 500, BK Medical, Denmark) and ultrasound probe (8818, BK Medical, Denmark) with 18G biopsy needles. Biopsies were performed in the axial plane by using the end-fire technique. A 6-core targeted biopsy was focused on the suspicious lesion as shown by mpMRI. The targeted biopsy was followed by 12-core standard systematic biopsy. If more than one suspicious lesion was identified on mpMRI, 3 cores from each of the two most suspicious lesions were sampled. There is no current consensus on the number of targeted cylinders. According to Mottet et al (3), at least 2 cores should be taken from the suspicious lesions. However, in many European urology centers, 4-6 cores are usually taken.

Before the cores were taken, patients received periprostatic block with 2% lidocaine (17). All patients received oral ciprofloxacin prophylaxis (18). The samples were examined by pathologists experienced in urological pathology to determine the Gleason score and percentage of core cancer involvement (19). The samples of 17 patients who underwent radical prostatectomy (RP) were pathohistologically evaluated.

### Statistical analysis

Descriptive statistics was used to describe patients' age, PSA, the number of previous biopsies, dimensions of the largest suspicious lesion on mpMRI, PI-RADS score, location of suspicious lesion on mpMRI, number of positive cores, the Gleason score in targeted and systematic biopsies, percentage of core cancer involvement of targeted and systematic cores, positivity of targeted and systematic biopsies, and clinical significance of prostate cancer.

The Wilcoxon signed rank test was used to compare the greatest percentage of cancer involvement and the Gleason scores between targeted and systematic cores. The McNemar test was used to compare the Gleason scores of targeted and systematic biopsies with the Gleason scores determined at pathohistological evaluation after RP. The level of statistical significance was set at less than 0.05. The analysis was conducted with SPSS, v. 25.0 software (IBM, Amonk, NY, USA).

## Results

The median age was 67 years (range, 57-84). In all patients, mpMRI was performed after at least one negative systematic biopsy using TRUS. The median number of biopsies that preceded mpMRI was 2 (range, 1-8). The median PSA value was 10.70 (range, 4.86-64.00) ng/mL. Digital rectal examination was unsuspicious for prostate cancer in all patients. The median size of the largest suspicious lesion on mpMRI was 1.30 cm (range, 0.4-3.00).

Overall prostate cancer detection, using either targeted or systematic biopsy, was 60.32% (38/63 patients). Systematic biopsies were positive in 30/63 patients (47.62%) and targeted biopsies in 33/63 patients (52.38%). The median number of positive cylinders in targeted biopsies was 4/6 (range, 0-6) and the median number of positive cylinders in systematic biopsies was 2/12 (range, 0-8). The median highest percentage of cancer involvement per biopsy core was 70% (range, 5%-90%) for targeted and 20% (range, 5%-90%) for systematic biopsy. According to the criteria by Klotz et al (GS≤6, clinical T1-2, PSA<10), the tumor found in systematic cores was low-risk in 8 patients (26.67%) and the tumor found in targeted cylinders was low risk in 6 patients (18.18%) (20). Prostate cancer was diagnosed in 8.33%, 45.71%, and 100% of PI-RADS 3, 4, and 5 lesions, respectively.

Thirty-six of 51 patients with PI-RADS 4 and 5 (70.59%) had prostate cancer in at least one cylinder, whether systematic or targeted. In 8 patients with PI-RADS 4 and 5 (15.69%), only targeted biopsy was positive, whereas in 4 patients (7.84%) only systematic biopsy was positive. On average, in patients with PI-RADS 4 and 5, there were 3 positive targeted (range, 0-6) and 2 positive systematic cylinders (range, 0-8).

Two of 12 patients with PI-RADS 3 lesion on mpMRI had positive biopsies. Targeted biopsy was positive in 1 patient and systematic biopsy in 2 patients (Table 1). The targeted biopsy had Gleason score 7, while both systematic biopsies had Gleason score 6.

**Table 1:** Biopsy data according to Prostate Imaging Reporting and Data System (PI-RADS) score

		Positive finding		Cancer core involvement	
PI-RADS score	Patients, n (%)	systematic biopsy, n (%)	targeted biopsy, n (%)	systematic biopsy (%)	targeted biopsy (%)
**3**	12/63 (19.05)	2/12 (16.67)	1/12 (8.33)	10	50
**4**	35/63 (55.56)	18/35 (51.43)	16/35 (45.71)	20	55
**5**	16/63 (25.40)	10/16 (62.50)	16/16 (100)	20	60

### Comparison between targeted and systematic biopsies

Targeted cores had significantly higher percentages of cancer core involvement than systematic cores (*P* = 0.003). Patients with positive systematic and targeted biopsy did not significantly differ in the Gleason score (*P* = 0.49). Targeted and systematic biopsies had an equal Gleason score in 19 cases. Systematic biopsies had a lower score in 5 cases and a higher score in 3 cases. In 17 patients in whom radical prostatectomy (RP) specimen was used as a control, systematic and targeted biopsy both matched the Gleason score of RP in 6 cases. Overall, targeted biopsy in more cases matched the Gleason score of RP specimen, but the difference was not significant (*P* = 0.219).

The majority of suspicious lesions were found in the transitional, anterior, and apical zones, and only 14.3% of lesions found using mpMRI were located in the peripheral zone (Table 2).

**Table 2:** Positivity of systematic and targeted biopsy according to lesion location on multiparametric magnetic resonance imaging (mpMRI)

**Lesion location on mpMRI**	Number (%)	Positive systematic biopsy, n (%)	Positive targeted biopsy, n (%)	Overall positivity, n (%)
**Central/transitional**	42/63 (66.67)	17/42 (40.48)	20/42 (47.62)	22/42 (52.38)
**Peripheral**	9/63 (14.29)	7/9 (77.78)	5/9 (55.56)	8/9 (88.89)
**Apical**	9/63 (14.29)	5/9 (55.56)	6/9 (66.67)	6/9 (66.67)
**Anterior**	3/63 (4.76)	1/3 (33.33)	2/3 (66.67)	2/3 (66.67)

## Discussion

In this study, the mpMRI performed before the repeated prostate biopsy helped in diagnosing prostate cancer in patients with a persistent clinical suspicion of prostate cancer due to elevated PSA values and present suspicious lesion on mpMRI but negative systematic biopsy.

There are three techniques of targeted prostate biopsy using the mpMRI: 1) cognitive MR imaging-targeted biopsy, 2) transrectal US-MR imaging fusion targeted biopsy 3) in-gantry MR imaging-targeted biopsy (21). Studies show that the three techniques do not significantly differ in the detection of clinically significant prostate cancer (10,22). In our study, we used cognitive MRI-targeted biopsy because it is a simple and quick technique that requires no new hardware, meaning there are no additional expenses for the hospital. However, the operator must be aware of the systemic biases caused by the fact that axial TRUS images are not obtained along the same plane as the MR images, although they look similar (23). This bias can lead to inaccurate targeting, particularly of the anterior and posterior lesions, and has to be taken into account when performing targeted biopsy with TRUS (24). The accuracy of this method highly depends on the operator’s understanding of the prostate MRI and the ability to correlate MRI targets to real-time TRUS images (15).

Despite the study limitations, primarily a highly selected subgroup of patients, certain conclusions can be made. We showed that neither systematic 12 core biopsy obtained using TRUS nor targeted biopsy using the cognitive fusion of mpMRI and TRUS technique can detect all cancers. The targeted biopsy was still somewhat superior to systematic biopsy, with a detection rate of 52.38% compared with 47.62%. However, the unexpectedly high positivity of systematic biopsy in our study could be explained by the fact that the operator, deliberately or not, directed the systemic biopsy toward the location of the suspicious lesion found on mpMRI. In 8 patients (15.69%), only targeted cores were positive and these findings are similar to the findings by Boesen et al (25).

A strength of our study is that we used pathohistological evaluation after RP as the control in 17 patients. Targeted biopsy undergraded prostate cancer in 6 cases (35.29%) and systematic biopsy undergraded it in 11 cases (64.71%), but the difference was not significant.

In our series, the prostate cancer detection rate using targeted and systematic approach based on mpMRI findings (60.31%) was higher than the detection rate found by Boesen et al (47%) and lower than that found by Delongchamps et al (67%) (25,26).

A low detection rate in patients with PIRADS 3, but high in those with PIRADS 5 lesions was consistent with the findings by Pokorny et al, indicating that patients with PIRADS 3 had a small benefit from a repeated biopsy (27).

If we had performed only targeted biopsy, we would have misdiagnosed 5 patients (13.16%), one of them with the Gleason score 8 on pathohistological evaluation after radical prostatectomy. This finding suggests that systematic biopsy is a necessary addition to targeted biopsy if we want to misdiagnose as few patients as possible (15,27-29).

The majority of suspicious lesions were located in the transitional, anterior, and apical zones, and only 14.3% of lesions found using mpMRI was were located in the peripheral zone This percentage was lower than in the study by Schouten et al (30%), possibly because they included more patients (30). The patients with lesions in the transitional, anterior, and apical zones had on average three previously negative biopsies because systematic biopsy is used for sampling primarily the peripheral zone. The use of end fire probe enabled the operator to reach the central and more apical portions of the prostate, otherwise inaccessible to sampling when using side fire probe for systematic 12-core biopsy.

We also showed that targeted biopsy matched the Gleason score of RP specimens more accurately than systemic biopsy, but without significant difference.

Our study demonstrated that the targeted approach based on mpMRI presented a valuable addition to systematic biopsy in diagnosing patients with repeated negative systematic biopsies but with constantly elevated or rising PSA. We expect that more experience in performing targeted mpMRI biopsies, as well as the development of mpMRI and targeted prostate biopsy techniques, will help us to perform only targeted repeat biopsies and omit systematic biopsy altogether.
